# Scientific Measurements in Sociology

**Published:** 1970-07

**Authors:** John Clutton-Brock

**Affiliations:** Professor of Anaesthetics, University of Bristol


### Scientific Measurements in Sociology

**John Clutton-Brock**

Professor of Anaesthetics, University of Bristol

Sociology is considered by many to be a somewhat inexact science; this may be because no clearly defined units of measurement in human relationships have been established.

Perhaps the most important of these relationships is that between men and women, so it is proposed briefly to suggest some clearly definable units in this field.

It has been previously suggested that the unit of female beauty should be the helen, sufficient beauty to launch a thousand ships. This may be inconveniently large today, particularly with the difficulties that exist in British shipyards, so the millihelen can be used as sufficient beauty to launch one ship. Smaller units, such as the microhelen or even the picohelen may occasionally be necessary.

There is, as yet, no unit of male beauty, but who better to represent this than Hermes, who, among his many exploits, managed to steal Aphrodites' girdle, surely no mean feat! The unit of male beauty would then be the herm or British Hermal Unit, B.H.U. One herm would be just sufficient to allow the seduction of one helen from a distance of one metre. The herm must not be confused with the therm which is merely a measure of the heat generated during the process.

It is not yet known whether the herm would obey the inverse square law as regards distance, because this work is seldom carried out under laboratory conditions. This point could surely soon be clarified, since the conventional Home Office licence is considered unnecessary today, either in the home or in the office, thanks to our permissive society.

Those wishing to do experimental work in this field, however, must be warned that unless adequate precautions are taken the following equation may well apply:

$$\frac{1 \text{ herm}}{1 \text{ helen}} = 1 \text{ preg.}$$

The unit of female resistance should perhaps be considered; as a courtesy one assumes that the resistance is female. However, this involves anti-body reactions and rejection phenomena, and a vast amount of work is at the moment going on in this field in relation to affairs of the heart and other organs, so it would be presumptuous to discuss this further at the present time.

Many other units of measurement will no doubt suggest themselves to the reader, and the author will be well satisfied if he arouses scientific interest in a subject which is only too often treated merely as a hobby.

(Reprinted from "Anaesthesia Points West" by permission of the Editor, and of the author).

